# Sphenopalatine Ganglion Blocks in Headache Management: A Review

**DOI:** 10.3390/brainsci15070672

**Published:** 2025-06-22

**Authors:** Andrei Lyle Bautista, Killian Coyne, Alexander Bautista, Alaa Abd-Elsayed

**Affiliations:** 1 School of Public Health and Information Sciences, University of Louisville, Louisville, KY 40202, USA; andrei.bautista@louisville.edu; 2 Division of Physical Medicine and Rehabilitation, University of Louisville, Louisville, KY 40202, USA; killian.coyne@louisville.edu; 3 Department of Anesthesiology and Perioperative Medicine, University of Louisville, Louisville, KY 40202, USA; 4 Chronic Pain Medicine, Department of Anesthesiology, University of Wisconsin-Madison, Madison, WI 53792, USA

**Keywords:** sphenopalatine ganglion, SPG block, headache, cluster headache, migraine, trigeminal neuralgia, facial pain, craniofacial pain, neuromodulation, refractory headache

## Abstract

Headache disorders are among the most prevalent and disabling neurological conditions worldwide, affecting more than three billion individuals and contributing to a substantial socioeconomic burden. Despite the availability of pharmacologic treatments such as triptans, NSAIDs, and CGRP monoclonal antibodies, a significant proportion of patients remain refractory or intolerant to these therapies. The sphenopalatine ganglion (SPG), a parasympathetic neural structure in the pterygopalatine fossa, is increasingly recognized as a critical node in the pathophysiology of primary headache disorders. SPG blocks—using local anesthetics, neurolytic agents, or electrical neuromodulation—offer a minimally invasive therapeutic approach by disrupting nociceptive transmission and autonomic activation. This narrative review synthesizes the anatomical and physiological rationale for SPG intervention, details various procedural techniques, evaluates clinical evidence across headache subtypes, and explores future research directions. Conditions covered include migraine, cluster headache, tension-type headache, trigeminal neuralgia, and persistent idiopathic facial pain. With expanding evidence and evolving technologies, SPG-targeted interventions have the potential to reshape the management of refractory headaches and facial pain syndromes.

## 1. Introduction

Headache disorders are among the most common neurological conditions, affecting up to 40% of the global population [1]. Migraine alone affects more than one billion people and is the second leading cause of years lived with disability globally [2]. Other primary headaches, such as tension-type (TTH) and cluster headache (CH), significantly impact quality of life and functionality. Despite advancements in acute and preventive pharmacologic treatments—including triptans, NSAIDs, CGRP antagonists, and anticonvulsants—many patients remain refractory or intolerant to standard care [3]. As defined by the European Headache Federation, refractory migraine refers to ≥8 debilitating headache days per month for ≥6 consecutive months, with failure of all available preventive drug classes due to either lack of efficacy or intolerable side effects [4].

This therapeutic gap has prompted renewed interest in neuromodulatory and interventional approaches. One such approach targets the sphenopalatine ganglion (SPG), a parasympathetic ganglion located in the pterygopalatine fossa and connected to the trigeminal and facial nerve pathways. The SPG modulates craniofacial nociception and parasympathetic outflow, making it a promising target for headaches with autonomic symptoms or trigeminal involvement [5].

Initially described by Sluder in 1908 in the context of “sphenopalatine neuralgia,” SPG intervention has evolved to include a variety of modern techniques such as transnasal anesthetic blocks, image-guided radiofrequency ablation, chemical neurolysis, onabotulinumtoxinA injections, and surgically implanted neurostimulation devices. Clinical applications now encompass a wide range of disorders, from migraine and CH to trigeminal neuralgia and persistent idiopathic facial pain (PIFP) [6,7].

This review consolidates anatomical and pathophysiological insights, procedural techniques, and clinical efficacy data to provide a comprehensive overview of SPG-targeted treatments in headache medicine. Literature was identified using keyword combinations such as “sphenopalatine ganglion block,” “SPG block,” “refractory migraine,” “cluster headache,” “treatment-resistant headache,” “SPG neurostimulation,” “facial pain,” and “chronic headache” across databases including PubMed, Scopus, and Google Scholar.

## 2. Anatomy and Pathophysiology

**Figure 1:**
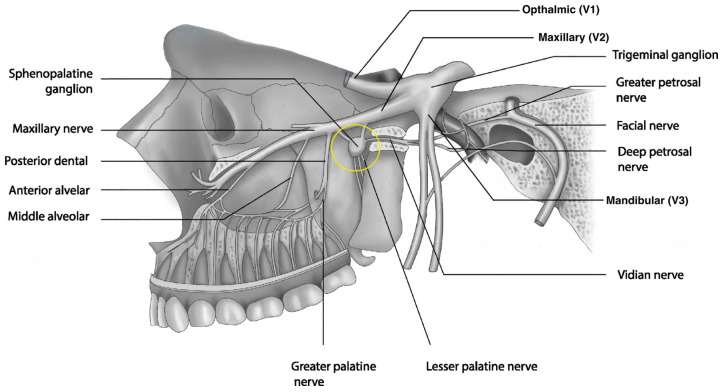
Anatomical location of the sphenopalatine ganglion within the pterygopalatine fossa and its surrounding structures. The sphenopalatine ganglion is circled in yellow. (Original figure created by the authors.)

The sphenopalatine ganglion (SPG), also called the pterygopalatine ganglion or Meckel’s ganglion, is the largest extracranial parasympathetic ganglion in the head [5]. It resides within the pterygopalatine fossa, posterior to the maxillary sinus and inferior to the foramen rotundum (Figure 1). This triangular space is bordered by the maxilla anteriorly, the palatine bone medially, and the pterygoid process posteriorly. The ganglion is suspended from the maxillary division of the trigeminal nerve (V2) by two pterygopalatine nerves and connects with several neural inputs:

- **Sensory afferents** from CN V2 pass through the ganglion without synapsing and provide sensation to the nasal cavity, nasopharynx, palate, and portions of the orbit and face. Additionally, due to its proximity to the ophthalmic (V1) and the mandibular (V3) divisions of the trigeminal nerve, there is an effect on V1 and a potential effect on V3 [8].
- **Preganglionic parasympathetic fibers** from the greater petrosal nerve (a branch of CN VII) synapse within the SPG and continue to innervate lacrimal, nasal, and pharyngeal glands.
- **Postganglionic sympathetic fibers** from the superior cervical ganglion reach the SPG via the deep petrosal nerve and travel through the ganglion to join other pathways without synapsing [6].

The SPG is a critical integration center between trigeminal nociceptive signaling and cranial autonomic responses. It is closely associated with the trigeminal-autonomic reflex, a loop in which trigeminal sensory input triggers parasympathetic output, leading to symptoms such as lacrimation, rhinorrhea, and nasal congestion. This reflex is especially relevant in trigeminal autonomic cephalalgias like cluster headache but also appears to play a role in migraine pathophysiology [9].

Evidence suggests that the SPG contributes to meningeal vasodilation and neurogenic inflammation by releasing vasoactive neuropeptides such as CGRP, substance P, and nitric oxide [10,11]. In migraine, parasympathetic activation from the SPG can amplify trigeminal sensitization and promote cortical spreading depression. In cluster headache, functional imaging studies have shown SPG activation during attacks, and its inhibition can terminate or reduce the severity of episodes [3].

### 2.1. Pathophysiology of Headache Disorders

Headache disorders such as migraines and cluster headaches involve hyperactivation of the trigeminovascular system. Triggers like hormonal fluctuations, stress, and specific foods activate sensory neurons, releasing pro-inflammatory neuropeptides, including calcitonin gene-related peptide (CGRP) and substance P. These molecules induce vasodilation, increase vascular permeability, and amplify pain signaling. 

SPG activation exacerbates these processes, contributing to the autonomic symptoms often associated with headaches (e.g., nasal congestion, lacrimation). SPG blocks interrupt these nociceptive pathways by inhibiting neurotransmitter release and attenuating neurogenic inflammation, providing therapeutic benefits.

### 2.2. Mechanisms of Action

The sphenopalatine ganglion (SPG) block works by interrupting pain pathways and modulating autonomic responses associated with headache disorders. Its mechanisms involve a combination of effects on nociceptive transmission, autonomic activity, and neurogenic inflammation, collectively contributing to pain relief.

#### 2.2.1. Inhibition of Nociceptive Transmission

The SPG serves as a critical relay point for sensory input from the maxillary division of the trigeminal nerve (CN V). During headache episodes, nociceptive signals from the trigeminal nerve are transmitted to the SPG, activating parasympathetic outflow and contributing to pain [3].

The block inhibits this sensory transmission by delivering local anesthetics or neurolytic agents to the SPG. This disruption prevents the further activation of central pain pathways, thereby reducing the intensity and duration of headaches. Furthermore, the block interrupts the trigeminal-autonomic reflex, a key mechanism in trigeminal autonomic cephalalgias like cluster headaches [9].

#### 2.2.2. Modulation of Autonomic Nervous System Activity

Because of the broad connections of the SPG with trigeminal and facial nerves, it mediates craniofacial autonomic symptoms such as lacrimation, rhinorrhea, and nasal congestion. These symptoms are hallmarks of conditions like cluster headaches and migraines.

SPG blocks suppress parasympathetic hyperactivity by interrupting the transmission of signals from preganglionic fibers of the facial nerve (CN VII) to postganglionic fibers that innervate the lacrimal and nasal glands. This modulation reduces autonomic symptoms that often accompany headache disorders, providing relief from pain and associated symptoms [7].

#### 2.2.3. Reduction in Neurogenic Inflammation

Headache disorders, particularly migraines, involve the release of pro-inflammatory neuropeptides, such as calcitonin gene-related peptide (CGRP) and substance P, from trigeminal nerve endings [10,11]. These neuropeptides lead to vasodilation, increased vascular permeability, and neurogenic inflammation, which amplify headache pain.

SPG blocks have been shown to reduce the release of these inflammatory mediators. By doing so, they attenuate the neurogenic inflammation and vascular changes that contribute to headache pathophysiology [12].

#### 2.2.4. Vasomotor Effects

The sympathetic fibers that pass through the SPG regulate craniofacial vasoconstriction. SPG blocks may help normalize vascular tone by modulating sympathetic activity, reducing the excessive vasodilation commonly seen during migraines and cluster headache attacks [13].

## 3. Techniques for SPG Blockade

Several techniques have been developed to block or modulate the SPG, ranging from non-invasive to percutaneous and surgically implanted modalities. The choice of approach depends on clinical context, duration of intended relief, provider expertise, and patient anatomy.

### 3.1. Transnasal Approach

The transnasal technique is the most commonly used approach due to its simplicity and minimal invasiveness. Local anesthetic (usually 4% lidocaine or 0.5% bupivacaine) is delivered to the posterior nasal cavity via the following:

- Cotton-tipped applicators;
- Flexible catheters;
- Commercially available devices such as Tx360^®^ or Sphenocath^®^.

This method targets the sphenopalatine foramen, approximating the SPG by capillary absorption and diffusion. Relief is often rapid, occurring within 15–30 min, but may be short-lived, lasting hours to a few days [14]. Repeated sessions are commonly required in chronic migraine or cluster headache.

### 3.2. Transoral Approach

This technique inserts a needle through the greater palatine foramen in the hard palate and advances superiorly into the pterygopalatine fossa under fluoroscopic or neuronavigation guidance. It allows direct anesthetic or neurolytic injection to the SPG. Though more invasive and requiring expertise, it offers deeper access and longer-lasting effects.

### 3.3. Percutaneous Infrazygomatic Approach

This image-guided approach involves inserting a needle under the zygomatic arch into the pterygopalatine fossa. It is most commonly used for the following:

- Radiofrequency ablation (RFA);
- Chemical neurolysis;
- Pulsed RFA.

Advanced imaging (fluoroscopy, CT, or ultrasound) ensures precise placement. This technique is typically reserved for patients with intractable headache or facial pain who have responded positively to diagnostic blocks.

### 3.4. Ultrasound-Guided Approach

The ultrasound-guided technique allows for real-time visualization of the SPG. By placing the ultrasound probe over the maxillary sinus, physicians can identify the pterygopalatine fossa and guide needle placement with improved accuracy. This approach enhances procedural safety and reduces the risk of complications associated with blind or fluoroscopy-guided techniques. It is gaining popularity due to its favorable safety profile and accessibility [15]. However, access to appropriate ultrasound equipment and a clinician skilled in regional anesthesia and facial anatomy is required.

## 4. Neurolytic Techniques

### 4.1. Radiofrequency Ablation

Radiofrequency ablation (RFA) involves the application of high-frequency electrical currents to generate thermal lesions in the SPG. While conventional RFA ablates nerve tissue directly, pulsed RFA offers a less destructive alternative that modulates pain pathways with a reduced risk of permanent damage. RFA is notably effective in managing cluster headaches, achieving approximately a 50% reduction in headache days while preserving sensory and motor function [16]. Nonetheless, the technique carries risks, including persistent numbness, neuroma formation, and accidental sensory nerve injury if precision is not maintained during needle placement.

### 4.2. Cryoablation

Cryoablation employs extreme cold to disrupt nerve conduction through Wallerian degeneration and apoptosis, providing a temporary yet effective interruption of parasympathetic outflow. While clinical data on SPG-targeted cryoablation for headache disorders are limited, this technique has demonstrated success in adjacent structures such as the posterior nasal nerve (PNN). A large cohort of patients with chronic rhinitis found that PNN cryoablation significantly reduced nasal symptoms and improved quality of life, with a favorable safety profile [17]. Given the shared autonomic pathways, extrapolation to the SPG is mechanistically reasonable. Reported side effects—including facial numbness and headache—are typically mild and self-limiting.

### 4.3. Chemical Neurolysis

Chemical neurolysis employs neurolytic agents such as ethanol and phenol to induce irreversible nerve damage through protein denaturation and axonal degeneration. This technique is typically reserved for severe, treatment-resistant cases, including trigeminal neuralgia and persistent idiopathic facial pain (PIFP). Directly disrupting nociceptive transmission within the SPG offers the potential for long-term symptom relief. However, this method carries notable risks, including neuropathic pain, soft tissue necrosis, and vascular injury, particularly if the agent spreads beyond the target zone [15,18].

### 4.4. CT-Guided Neurolysis

CT-guided neurolysis involves the precise delivery of neurolytic agents, such as oxygen-ozone gas or alcohol-based compounds, to both the sphenopalatine ganglion (SPG) and trigeminal ganglion (TG) to treat persistent idiopathic facial pain (PIFP). This dual-target approach has demonstrated superior clinical outcomes compared to SPG neurolysis alone, particularly in patients exhibiting neuropathic features, such as burning or electric shock-like pain [15]. The combination allows for more comprehensive disruption of craniofacial pain circuits while maintaining a minimally invasive profile. Although promising, the procedure carries risks, including facial hematoma, epistaxis, transient sensory disturbance, and, in rare cases, needle misplacement through the lateral nasal wall when using the infrazygomatic arch approach. Moreover, retrospective designs and small sample sizes limit the current evidence base, underscoring the need for high-quality prospective trials to validate their long-term efficacy and safety.

## 5. Botulinum Toxin (Botox) SPG Injections

Botulinum toxin injections targeting the sphenopalatine ganglion (SPG) modulate pain pathways by inhibiting the release of acetylcholine at cholinergic terminals, promoting local neuromuscular and parasympathetic inhibition. This neuromodulatory effect disrupts nociceptive signal transmission and autonomic overactivity, offering therapeutic benefits in selected patients. Limited clinical studies have reported substantial reductions in both pain intensity and attack frequency in patients with chronic migraine, chronic cluster headache, and trigeminal neuralgia following SPG-targeted onabotulinumtoxinA injections [14,19,20,21]. Advantages include its non-invasive nature, effectiveness in refractory cases, and potential for long-term symptom control with periodic administration. However, transient adverse effects such as hemifacial weakness, localized bruising, or injection-site discomfort have been noted—likely due to spread to adjacent facial nerve branches. Additionally, repeat injections are typically required to maintain efficacy, posing logistical and financial challenges for some patients.

## 6. Patient Self-Administered SPG Blockade

Another promising yet controversial avenue is the expansion of SPG blocks as an at-home treatment modality. Proponents argue that enabling early, self-administered intervention during prodromal or acute headache phases could improve efficacy, reduce emergency visits, and alleviate healthcare system burdens. However, this approach raises several concerns. Patient-administered blocks may increase the risk of improper application, adverse effects such as epistaxis or anesthetic allergy, and reduced procedural sterility. Legal liability, lack of training, and variable patient anatomy further complicate implementation. Additionally, without direct supervision, distinguishing between headache subtypes or recognizing red-flag symptoms may be challenging. While at-home protocols using nasal spray formulations or simplified delivery devices warrant further exploration, large-scale trials evaluating safety, patient adherence, and long-term outcomes are essential before such strategies can be widely adopted [22,23].

## 7. Neuromodulation

Neuromodulation techniques—including sphenopalatine ganglion (SPG) stimulation and peripheral nerve stimulation (PNS)—offer promising options for patients with refractory headache disorders who have not responded to pharmacologic or injection-based therapies.

### 7.1. SPG Stimulation

Sphenopalatine ganglion (SPG) stimulation involves the surgical implantation of a miniaturized wireless neurostimulator into the pterygopalatine fossa, typically via a transoral approach with fixation to the posterior maxilla. The patient externally activates the device using a cheek-mounted remote control, delivering targeted electrical impulses to disrupt trigeminal-autonomic signaling pathways implicated in cluster headache and craniofacial pain. The technique is minimally invasive, fully reversible, and offers acute and preventive therapeutic effects.

In the multicenter, randomized, sham-controlled Pathway CH-1 trial, SPG stimulation aborted 67% of cluster headache attacks compared to 7% in the sham group, with many patients also reporting reduced attack frequency and improved quality of life [24]. A model-based economic analysis based on this trial projected favorable cost-effectiveness, driven by reduced medication use and improved patient outcomes [25]. The trial also reported that 64% of participants experienced acute relief from chronic cluster headache attacks within 15 min of SPG stimulation, and 43% showed ≥50% reduction in attack frequency [24]. Long-term data show that over 60% of patients maintained therapeutic response at 24 months [26].

Long-term data from a 24-month observational follow-up study further support the sustained efficacy of SPG stimulation. Jürgens et al. (2017) reported that over 80% of patients experienced a therapeutic response of at least 30%, with most responders achieving a ≥75% reduction in attack frequency, classifying them as extreme responders to SPG neuromodulation [7,26].

SPG stimulation is CE-marked in Europe but remains investigational in the United States. The implant procedure is minimally invasive but carries risks, most commonly transient sensory disturbances such as numbness in the distribution of the maxillary nerve, facial swelling, or pain. These side effects are typically mild and resolve within three months. Rare complications, such as lead migration, have been reported. Additionally, the phenomenon of “side shift,” in which cluster attacks migrate to the contralateral side post-implantation, has been described. Although SPG stimulation has not been studied in pediatric, pregnant, or lactating populations, bilateral implantation may offer a future option for patients with side-alternating attacks. Further research is necessary to clarify its long-term efficacy, safety, and broader clinical applicability [26,27,28].

The Pulsante^®^ microstimulator is the only FDA IDE-cleared implantable device specifically designed for SPG neuromodulation. Developed by Autonomic Technologies, Inc., it is anchored to the maxilla and delivers stimulation via a handheld remote.

Implantation requires careful preoperative imaging to assess the anatomy of the pterygopalatine fossa (PPF). Contraindications include active midfacial infection, recent facial surgery or radiation, and severe maxillary bone loss [26]. Adverse effects are typically mild, with most cases of sensory disturbance resolving within 3 months. The device may reduce reliance on abortive medications and has demonstrated cost-effectiveness in long-term modeling studies [29].

### 7.2. Peripheral Nerve Stimulation (PNS)

Peripheral nerve stimulation (PNS) targets extracranial nerves—most commonly the greater and lesser occipital nerves—using subcutaneously implanted electrodes that deliver low-frequency impulses. It is primarily used for chronic migraine and cluster headache, especially when symptoms extend beyond the SPG’s innervation territory. PNS may also play a role in patients with occipital tenderness, cervicogenic overlap, or scalp allodynia.

However, clinical evidence supporting the efficacy of PNS remains limited and mixed. Notably, the ONSTIM trial, a randomized controlled study evaluating occipital nerve stimulation in chronic migraine, reported modest reductions in headache days but failed to achieve its primary efficacy endpoint, and the placebo response was high [30]. Other studies, including the PRISM trial and various case series, have demonstrated inconsistent outcomes and high device-related complication rates, including lead migration, infection, and hardware malfunction [31,32].

Long-term outcome data are sparse, and heterogeneity in lead placement, stimulation parameters, and patient selection further complicates interpretation. Additionally, there is no FDA-approved indication for headache-related PNS, and reimbursement barriers remain substantial. Given these limitations, PNS should be reserved for highly selected patients with refractory symptoms and well-documented failure of more conservative treatments, ideally within clinical trials or specialized centers.

## 8. Evidence for Other Headache and Facial Pain Syndromes

### 8.1. Tension-Type Headache (TTH)

Unlike migraine or cluster headache, TTH lacks significant autonomic features and is primarily thought to arise from pericranial muscle tension and central sensitization. As such, the rationale for SPG involvement is weak, and clinical use is minimal. No randomized controlled trials (RCTs) or large case series currently support using SPG blocks [2]. Some experts hypothesize a minor role for SPG block if coexistent trigeminal sensitization is present, but this remains unproven.

Patients with chronic daily headache or transformed migraine may benefit more from occipital nerve blocks, trigger point injections, or pharmacologic preventive therapy than SPG interventions.

### 8.2. Trigeminal Neuralgia (TN)

Trigeminal neuralgia is characterized by lancinating, paroxysmal pain in the CN V1–V3 distribution, often triggered by light stimuli. While primary interventions include carbamazepine, microvascular decompression, or glycerol rhizotomy, some patients—especially those with maxillary (V2) pain—have responded to SPG-targeted therapies.

Recent studies demonstrate promising results for SPG Botox injection in TN. A prospective observational study on 14 patients with refractory TN who received 40 units of onabotulinumtoxinA per side to the SPG showed a 50% reduction in pain scores and daily attack frequency by 60 days. Though transient hemifacial weakness occurred in 77% of patients, all cases resolved within three months [21].

SPG blocks may modulate sensory inflow and autonomic coactivation associated with TN, especially when V2 or “atypical” TN symptoms are predominant [21]. SPG neurolysis has also been used historically, albeit with greater risk.

### 8.3. Persistent Idiopathic Facial Pain (PIFP)

PIFP is a chronic pain condition localized to the face, often lacking precise nerve territory distribution or structural etiology. It has overlapping features with atypical facial pain and can be highly refractory.

SPG intervention is gaining traction in this space, especially when neuropathic features (burning, sharp pain) are present. A study involving CT-guided neurolysis of the SPG and trigeminal ganglion (TG) in 34 patients with PIFP found that dual-target neurolysis resulted in significantly higher rates of ≥50% pain relief at one month compared to SPG-only blocks. Patients with positive neuropathic screening (e.g., DN4 > 4) responded better [15].

This suggests that selective patient screening may improve success rates, and targeting multiple cranial pain pathways may be necessary for complex facial pain syndromes. The safety profile was acceptable, with only transient sensory disturbances reported.

## 9. Systematic Reviews and Meta-Analyses

Given the small size of many individual studies, systematic reviews help clarify the overall value of SPG interventions:

- Mojica et al. (2017) conducted a narrative review of the SPG block for chronic headaches. They found consistent short-term relief for migraine and cluster headache, although variability in technique and outcome measures limited pooled analysis [33].
- Sánchez-Gómez et al. (2021) reviewed clinical trials on SPG neurostimulation, concluding it is a safe and effective therapy for chronic cluster headache. While adverse events (mostly minor) were common in the immediate postoperative period, most resolved without intervention [34].
- Láinez et al. (2014) conducted a randomized, sham-controlled trial to evaluate sphenopalatine ganglion (SPG) stimulation for chronic cluster headache. Among 32 implanted patients, 68% experienced an acute or preventive response. SPG stimulation led to a 67% rate of pain relief in treated attacks compared to 7% with sham, and over 40% of patients achieved a ≥50% reduction in attack frequency. Most adverse events were mild and related to the implantation procedure, supporting the safety and effectiveness of SPG stimulation in this population [35].
- Ho et al. (2017) systematically reviewed SPG block, radiofrequency ablation (RFA), and neurostimulation across 83 studies. The most substantial evidence supported all three modalities for cluster headache, with moderate evidence for SPG blocks in trigeminal neuralgia, migraine, and postoperative sinus pain. Evidence for other conditions, such as PIFP, postherpetic neuralgia, and cancer pain, was limited to case reports. SPG RFA showed longer-lasting relief than blocks, while neurostimulation demonstrated significant acute and preventive efficacy in chronic cluster headache [36].

Limitations of these reviews include the predominance of small-scale studies, such as case series and single-center trials, with only a few randomized controlled trials (RCTs) available, most notably in cluster headache. There is significant heterogeneity in procedural techniques (e.g., transnasal cotton swabs vs. image-guided infrazygomatic injections), intervention types (e.g., local anesthetics, radiofrequency ablation, botulinum toxin, neurostimulation), and outcome measures (e.g., pain intensity, frequency, quality of life), making direct comparisons difficult and limiting the strength of pooled conclusions. Additionally, many conditions beyond cluster headache, such as trigeminal neuralgia, migraine, and PIFP, are supported primarily by lower-level evidence, including case series or small observational cohorts. Ho et al. (2017) highlighted that even where promising effects were observed, most studies lacked replication and robust methodology, highlighting the need for larger, standardized, multicenter RCTs to confirm efficacy and define optimal patient selection criteria [36].

## 10. Contraindications

Sphenopalatine ganglion (SPG) interventions, including blocks, radiofrequency ablation (RFA), and neurostimulation, are generally safe but require careful patient screening. The following contraindications should be considered when evaluating candidates for SPG-targeted procedures:

**Absolute contraindications** include the following [37,38]:

- Patient refusal or inability to provide informed consent.
- Known allergy to local anesthetics or chemical neurolytic agents (e.g., ethanol, phenol).
- Active infection at the site of injection or systemic infection.
- Uncorrected coagulopathy or ongoing anticoagulation therapy, particularly when procedures involve non-compressible or vascular-adjacent areas.
- Presence of a pacemaker (in the case of planned RFA) without prior consultation with cardiac electrophysiology for device interrogation and safety clearance.

**Relative contraindications** include the following [37,38]:

- Anatomical barriers such as severe nasal septal deviation, facial trauma, or obstructive polyposis that may impede access (especially for transnasal techniques).
- Preexisting neurologic deficits in the distribution of the maxillary nerve (CN V2), which may confound post-procedural assessments.
- Psychological factors such as severe procedural anxiety or inability to tolerate awake interventions.
- Recent midfacial surgery or implanted hardware that increases risk during needle-based or surgical approaches.

Diagnostic lidocaine blocks may help predict response and tolerability in borderline cases, especially before more invasive neurolytic or neuromodulatory treatments [37,38].

## 11. Economic Evaluations

Cost-effectiveness is critical in adopting SPG-targeted interventions, particularly given the high procedural costs associated with neurostimulation and ablative techniques. While short-term relief can often be achieved with anesthetic blocks, longer-lasting modalities such as SPG stimulation and radiofrequency ablation (RFA) entail significant upfront investment.

A landmark model-based analysis by Pietzsch et al. (2015), using data from the Pathway CH-1 trial, concluded that SPG stimulation for chronic cluster headache (cCH) yields an incremental cost-effectiveness ratio (ICER) of EUR 2736 per quality-adjusted life year (QALY) gained over 5 years in the German healthcare system, well below typical willingness-to-pay thresholds [25]. The primary drivers of cost savings were reduced reliance on abortive medications (e.g., triptans, oxygen), fewer healthcare encounters, and improvements in health-related quality of life (HRQoL), particularly among patients with ≥3 attacks per week.

Although robust economic analyses for SPG RFA and blocks are lacking, the procedures are less resource-intensive than neurostimulation and may offer favorable short- to medium-term cost profiles. Successful RFA has been associated with decreased medication burden and reduced need for follow-up visits in refractory headache patients, but published cost analyses remain limited.

Moreover, the cost-effectiveness of SPG interventions scales with attack frequency and refractoriness. Patients with chronic or high-frequency episodic headache, who frequently use triptans or visit the ER, stand to benefit most from interventions that can reduce their medication dependence and healthcare utilization.

Future research should focus on direct head-to-head comparisons of SPG procedures with pharmacologic and other interventional treatments, incorporating real-world data on medication use, ER visits, and quality-adjusted survival.

## 12. Future Directions

As SPG interventions gain broader acceptance in headache management, several important areas for further research and innovation are necessary. Future directions for SPG interventions in headache management include the need for large, high-quality, randomized controlled trials comparing SPG blocks to sham interventions, pharmacologic treatments, and other procedures like occipital nerve blocks or trigeminal decompression while evaluating optimal dosing, frequency, anesthetic agents, placebo effect, and patient selection criteria such as CGRP responsiveness. Additionally, further research on patient self-administered techniques is necessary for further adoption. Research on predictive biomarkers—such as CGRP, substance P, VIP levels, SPG imaging findings, DN4 or HIT-6 scores, and response to diagnostic lidocaine blocks—may enable more personalized, cost-effective care. Technological innovations like ultrasound-guided or navigation-assisted delivery, 3D-printed Botox guides, extended-release formulations, nanoparticle systems, and non-invasive stimulation could enhance precision, duration, and accessibility [20]. Combining SPG interventions with CGRP monoclonal antibodies, occipital or peripheral trigeminal nerve treatments, behavioral therapies, or CBT may yield synergistic benefits by targeting both central and peripheral mechanisms of head pain.

## 13. Limitations and Safety Considerations

Despite their promise, SPG-targeted treatments have limitations, including the short duration of effect from most local anesthetic blocks, risks associated with invasive techniques such as bleeding, numbness, or infection, and the off-label use of neurotoxins or chemical neurolysis agents. Additional challenges include limited access, reimbursement barriers—particularly for neuromodulation—and variable patient responses that complicate outcome prediction. However, most reported adverse effects are mild and transient, especially with transnasal or ultrasound-guided approaches, and serious complications remain rare when proper technique is employed.

## 14. Conclusions

Sphenopalatine ganglion (SPG) interventions represent a rapidly evolving frontier in the management of refractory headache and facial pain syndromes. The SPG serves as a unique anatomical and physiological target due to its intersection of trigeminal sensory pathways and parasympathetic output. Interventions that modulate or block SPG activity—whether via transnasal anesthetic application, percutaneous neurolysis, botulinum toxin injection, or neuromodulation—offer effective options for patients who fail to respond to conventional treatments.

The most robust data support SPG interventions in cluster headache and migraine, particularly in chronic and medication-refractory forms. Emerging evidence also supports their use in trigeminal neuralgia, persistent idiopathic facial pain, and select facial pain disorders. Techniques continue to evolve, with advances in guidance, safety, and duration of relief.

## Data Availability

No new data were created in this study. The figure included was created by the authors for illustrative purposes.
